# Application of modified multi-verse optimization for temperature control in thermal power plant condensers

**DOI:** 10.1038/s41598-026-40559-7

**Published:** 2026-03-06

**Authors:** Sweta Panda, Soumya Ranjan Das, Arun Kumar Sahoo, M. UmaMaheswar Rao, Norah Saleh Alghamdi, Wattana Viriyasitavat, Gaurav Dhiman

**Affiliations:** 1 Department of Electrical Engineering, VSSUT Burla, Burla, Odisha India; 2 Manipal Institute of Technology, Manipal Academy of Higher Education, Manipal, India; 3 Department of Electrical Engineering, IIIT Bhubaneswar, Bhubaneswar, Odisha India; 4 Department of CGDT/ Design, IIT Hyderabad, Hyderabad, India; 5 Department of Computer Sciences, Princess Nourah Bint Abdulrahman University, Riyadh, Saudi Arabia; 6 Department of Statistics, Chulalongkorn University, Bangkok, Thailand; 7 Department of Computer Science and Engineering, Yuan Ze University, Tao Yuan, Taiwan; 8 Centre of Research Impact and Outcome, Chitkara University, Punjab 140417 Rajpura, India

**Keywords:** Surface condenser, PID controller tuning, GA, MVO, Energy science and technology, Engineering, Mathematics and computing

## Abstract

The surface condenser exhibits nonlinear dynamics and inherent time delays, making precise temperature regulation essential for stable operation in thermal power systems. In this study, a Modified Multi-Verse Optimizer (MMVO) is employed to tune the parameters of a PID controller for improved temperature control of a shell-and-tube condenser. The methodology involves formulating the PID tuning task as an optimization problem, applying MMVO with defined search bounds, and evaluating its performance using 23 standard benchmark functions and repeated simulation runs. Statistical indicators including best, worst, average, and standard deviation values across 30 independent executions are used to assess robustness. Comparative analyses with Ziegler–Nichols (ZN), Genetic Algorithm (GA), and the original Multi-Verse Optimizer (MVO) demonstrate that the modified approach achieves lower integral error indices and reduced overshoot, while providing more consistent performance across trials. The results indicate that MMVO-based PID tuning offers enhanced control capability for systems with strong nonlinearities and delay characteristics.

## Introduction

Currently, surface condensers are commonly used in thermal power plants, processing plants, food production, oil refineries, and mechanical systems for the transfer of thermal power. The heat transfer process occurs within shell and tube heat exchangers. A surface condenser^1,2^ typically converts steam from a gaseous form to a liquid form below the atmospheric pressure. In industrial settings, large heat exchanger^3^ systems are employed to harness any wasted thermal energy. Shell and tube heat exchangers^4,5^ are favored in industries due to their higher efficiency. The heat exchanger is a complex system characterized by high non-linearity and delay time that affect its working conditions. These time delays can also create undesired effects on the system, potentially resulting in instability. Consequently, the primary goal is to maintain the temperature of the process fluid at a specified set point that meets process requirements by regulating the control valve. Therefore, a robust method is essential to achieve optimal function from the heat exchanger.

Initially, several studies have been conducted to obtain optimal response from heat exchangers^6–9^. Some traditional techniques, such as the PID controller, have been discussed in^10^. The parameters for these techniques are adjusted using the Z-N tuning approach^11,12^. However, the response still exhibits significant overshoot and requires more time to stabilize. To further reduce both overshoot and settling time, the PID controller is fine-tuned using the guaranteed dominant pole placement method^13,14^. Tuning the PID controller via the guaranteed dominant pole placement reduces overshoot but introduces a steady-state error in the process. In the past ten years, several nature-inspired algorithms^15,16^ have been effectively developed to address these challenges. By incorporating optimization techniques into the system, we can achieve results that are closer to the ideal response. Some commonly used evolutionary algorithms include the GA^17,18^, Particle Swarm Optimization (PSO)^19^, and Moth Flame Optimization (MFO)^20,21^.

Recent studies have highlighted the increasing use of advanced metaheuristic algorithms in optimization and control applications. In^22^, the authors have proposed the modified atom search optimizer which combines an adaptive gbest-guided mechanism to overcome weaknesses in the standard atom search optimizer. Through this proposed optimizer, time wastes that are related to poor local optimum and loss of control over exploration and exploitation processes are tamed, meaning that this idea is suited for difficult instances of the optimizer. Similar to this, the author of^23^ suggests a novel metaheuristic-driven control method for temperature regulation of the continuous stirred-tank heater process by integrating the recently created starfish optimization algorithm with the two degrees of freedom-PID acceleration control. The combination of both gives a versatile and efficient technique for managing highly nonlinear systems, with important implications for industrial temperature regulation applications. In^24^, an integrated approach featuring a proportional–integral–derivative with N filter (PIDN) controller alongside the artificial rabbit’s optimization algorithm to enhance temperature control in electric furnaces, addressing key challenges in precision and response stability. The proposed PIDN controller incorporates adaptive tuning techniques designed to improve response accuracy and reduce overshoot, tailored specifically for the dynamic requirements of electric furnace applications. In^25^, the diligent crow search method is utilized to optimize a novel multistage controller, TDn (1 + PIDn). To handle system nonlinearities, external disturbances, and the intricacies of dynamic reactions in steam condensers, the suggested controller was created. To increase modeling accuracy, stability, and robustness, the current work builds on existing developments by introducing a modified Atom Search Optimizer specifically designed for IIR system identification^26^. These works collectively underline the usefulness of contemporary optimization methods in addressing complicated control and signal-processing difficulties.

This paper focuses on optimizing PID controller parameters using GA and MVO algorithms. However, these algorithms can become trapped in local minima. Although various metaheuristic algorithms have been applied to PID tuning, the literature shows limited attention to optimization methods specifically adapted to the nonlinear and time-delay characteristics of surface condensers. Most existing studies rely on standard GA, PSO, or MVO approaches, which often face convergence issues and local-optima constraints in such systems. This gap highlights the need for a modified optimization strategy capable of improving tuning performance under these challenging dynamic conditions.

First, a modified Multi-Verse Optimizer (MMVO) is formulated by altering the exploration and exploitation mechanism to improve performance in nonlinear and time-delay systems. Second, this modified optimizer is employed for tuning PID control parameters specifically for a surface condenser, a configuration that has received limited attention in prior optimization-based studies. Third, a comprehensive evaluation is performed using 23 benchmark functions and repeated simulation runs to assess robustness, with statistical indicators reported. Finally, the proposed tuning method is compared with Ziegler–Nichols, GA, and the original MVO to demonstrate consistent improvements in overshoot, settling behavior, and integral error metrics.

The main objective in this research is:

1. To maintain the temperature of the outgoing fluid of the shell and tube heat exchanger at a desired set point by adjusting steam flow rate using the PID controller.
2. To overcome the problems associated with GA and MVO.
3. To implement Modified Multi Verse Optimizer technique to optimize the PID controller parameters.
4. To justify the efficiency of the proposed algorithm.
5. To study the time response analysis of the process.

The paper is organized as follows. Section 2 presents the mathematical model of the surface condenser and its process components. Section 3 introduces the PID controller and the tuning approaches used. Section 4 describes the Modified Multi-Verse Optimizer (MMVO) and its procedure. Section 5 outlines the benchmark setup, simulation framework, and statistical criteria. Section 6 discusses the comparative results, and Sect. 7 concludes the study with future research directions.

## Heat exchanger

**Fig. 1:**
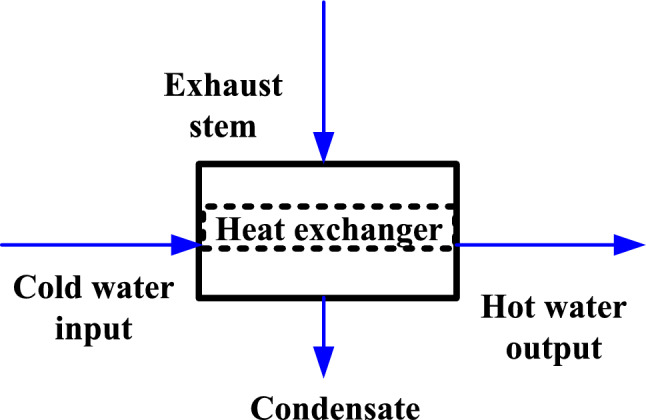
Principle of heat exchanger.

Almost all industrial facilities comprise the creation or inclusion of energy in the way of heat. In process industries, heat exchangers are basically employed for heat transfer from one fluid to another through a solid barrier^1^. They are primarily used in power plants for recovering waste heat. Various kinds of heat exchangers are utilized in industrial usages, but shell and tube heat exchangers are the most prevalent and are able to deal a broad range of temperatures and pressures^2^. These exchangers offer a greater surface area for heat transfer and can be manufactured in diverse sizes and configurations more easily (Fig. 1).

### Mathematical model

Different assumptions are made while establishing the mathematical model:

- $$Rate of Inflow_{fluid} \, = Rate of Outflow_{fluid}$$ i.e., $$Height_{fluid} = fixed$$
- Heat loss to the atmosphere is negligible.
- The physical properties density ($\rho$), latent heat of vaporization of steam ($\lambda$) and specific heat (${C}_{p}$) do not vary significantly with temperature.

### Mass balance equation

Rate of mass accumulation within the tank:$\frac{{d{ rho }V}}{dt}$.

Mass balance equation is

1 $${\rho Fi}-{\rho F }= \frac{d \uprho V}{dt}$$

As rate of mass input to the tank = rate of mass output to the tank and density is constant

$$\frac{dV}{dt}=0$$

V = Constant

Thus, mass balance equation concludes that system have a constant volume.

### Energy balance equation

For this system, total energy is the sum of kinetic energy, internal energy and Potential energy. Rate of accumulation of energy only involves the internal energy.
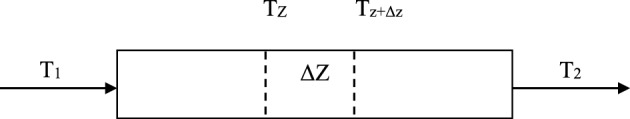


Liquid temperature changes with time along the axial direction z from the value T_1_ at the inlet to value T_2_ at the exit. It is a distributed parameter system.

### Energy balance equation with control volume AΔz

The buildup of enthalpy over the time interval Δt is equal to the enthalpy entering during Δt minus the enthalpy exiting during Δt, plus the enthalpy transferred from the steam to the liquid during Δt.

2 $$\begin{aligned} \rho C_{P} A\Delta z[T_{t + \Delta t} {-}T_{t} ] \, & = \rho C_{P} \nu A \, T_{Z} \Delta t \, - \rho C_{P} \nu A \, T_{Z + \Delta Z} \Delta t + Q\Delta t \, (\pi D\Delta z) \\ \rho C_{P} A\frac{\partial T}{{\partial t}} + \rho C_{P} \nu \frac{\partial T}{{\partial Z}} & = Q \, \pi D \\ \end{aligned}$$

Hence, temperature is the dependent variable and function of two independent variable (t, Z).

## System investigated

The system under investigation consists of a heat exchanger system and chemical reactor. The storage container contains the fluid from the chemical reactor, which is then pumped to the heat exchanger where it is heated to a specified temperature by the boiler. The storage tank delivers the processed fluid to the heat exchanger system through a pump and a one-way valve. In this setup, steam flows through the tubes. After the steam transfers heat to the process fluid, the condensed steam exits the heat transfer system at 100℃.

Various hypotheses have been proposed to mathematically represent the heat exchanger system. The following are enumerated below.

3 $$Rate \;of\;Inflow_{fluid} = Rate \;of\;Outflow_{fluid} \;\;\;i.e.\;\;\;Height_{fluid} = fixed$$

Heat storing capacity is immaterial.

**Fig. 2:**
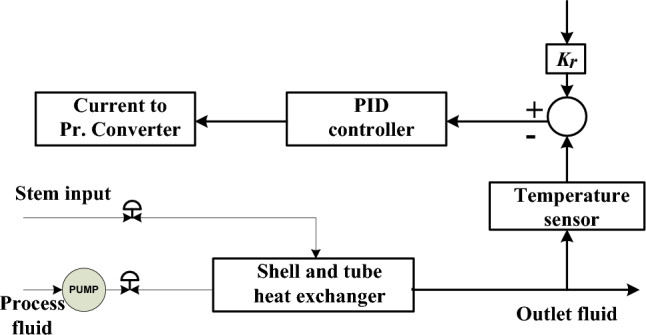
Schematic diagram of temperature control of heat exchanger system.

In this control loop for feedback process, the controller functions in a reverse-acting manner, while the valve operates on an air-to-open (fail-close) basis. Figure 2 illustrates a schematic figure of temperature control within the heat exchanger system.

## Experimental data

The components of the heat exchanger system, including the valve, actuator, and sensor, have been mathematically represented based on the experimental data summarized in Table 1.

**Table 1:** Parameter of process data^27^.

Parameter	Value
Surface condenser reaction to the steam flow gain	$50^\circ{\rm C}$/(kg/sec)
Time constants	$30\mathrm{sec}$
Surface condenser reaction to deviation of process fluid flow gain	$1^\circ{\rm C}$/(kg/sec)
Surface condenser reaction to deviation of process temperature gain	$3^\circ{\rm C}$/°C
Actuator capacity	$1.6$ kg/sec of steam
Actuator time constant	$3\mathrm{sec}$
Temperature sensor variation	50 °C to150°C
Temperature sensor time constant	10 s

## Controller and optimization techniques

### PID controller

A PID controller was selected for this study because it represents the most widely adopted control strategy in process and thermal systems, including surface condensers. Its simplicity, ease of tuning, and strong disturbance-rejection capability makes it suitable for processes exhibiting nonlinear behavior and time delays. Moreover, PID-based regulation aligns with current industrial practice, enabling direct applicability of the optimized parameters without requiring additional hardware or structural modifications. The control signal is derived as using PID control is:

4 $$u\left( t \right) = {K}_{{c}} ({e}({t}) + \frac{1}{{\tau_{i} }}\mathop \smallint \limits_{o}^{t} e(t) + \tau_{d} \frac{{{de}({t})}}{{{dt}}})$$

where, e(t) is the error signal, u(t) is the controller output, K_c_ is the controller gain, τ_i_ and τ_d_ are integral gain and derivative gain respectively.

The PID controller can be represented in Laplace domain as

5 $${\mathrm{G}}_{{{\mathrm{PID}}}} \left( {\mathrm{s}} \right) = \frac{{{u}({s})}}{{{e}({s})}} = {K}_{{c}} \left( {\frac{{{ tau }_{d} { tau }_{i} S^{2} + { tau }_{i } S + 1}}{{{ tau }_{i } S}}} \right)$$

The equation for real PID controller is denoted as

6 $${\mathrm{G}}_{{{\mathrm{PID}}}} \left( {\mathrm{s}} \right) = \frac{{{u}({s})}}{{{e}({s})}} = K_{c} \left( {\frac{{{ tau }_{i } S + 1}}{{{ tau }_{i } S}}} \right)\left( {\frac{{{ tau }_{d } S + 1}}{{1 + { tau }_{f } S}}} \right)$$

Here ${\tau }_{f}$ indicates the filter parameter. Equation (6) is narrated as:

7 $${\mathrm{G}}_{{{\mathrm{PID}}}} \left( {\mathrm{s}} \right) = \frac{{{u}({s})}}{{{e}({s})}} = K_{c} \left( {\frac{{{ tau }_{i } S + 1}}{{{ tau }_{i } S}}} \right)\left( {\frac{{{ tau }_{d } S + 1}}{{1 + { alpha \tau }_{d } S}}} \right)$$

**Fig. 3:**
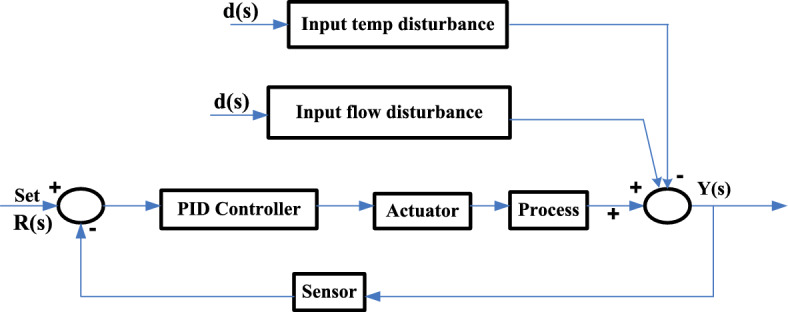
Block diagram of feedback control loop.

By substituting ${\tau }_{f}$ = α ${\tau }_{d}$ Here α is the filter coefficient (Fig. 3).

### Tuning of PID controller

There are several traditional techniques for tuning a PID controller. One such technique is the Ziegler-Nichols (ZN) method. In this approach, gain is referred to as the critical gain ${\mathrm{k}}_{\mathrm{cu}}$. A drawback of the ZN method is that it tends to produce a significant maximum overshoot (Table 2).

**Table 2:** Different closed loop oscillation based tuning methods.

Type of tuning methods	${k}_{c}$	${\tau }_{i}$	${\tau }_{d}$
Z-N	$0.6{\mathrm{k}}_{\mathrm{cu}}$	$0.5\mathrm{T}$	$0.125\mathrm{T}$
Tyreus-Luyben	$0.45{\mathrm{k}}_{\mathrm{cu}}$	$2.2\mathrm{T}$	$0.15\mathrm{T}$

The characteristic equation $(1+\mathrm{G}(\mathrm{s})\mathrm{H}(\mathrm{s})$=0) is represented as:

8 $$900{s}^{3} + 420{s}^{3} + 43{s} + 0.798{ k}_{{{cu}}} + 1 = 0$$

By implementing Routh stability in Eq. (8) gives ${k}_{{{cu}}} = 23.8$

The Auxiliary equation is:

9 $$420{s}^{2} + 0.798{ k}_{{{cu}}} + 1 = 0$$

By replacing $s=j\omega$ in Eq. (9), gives $\omega = 0.218 \;and\; T = 28.79$.

#### Z-N tuning method

The PID controller parameters (${\mathrm{k}}_{\mathrm{c}}$, ${\uptau }_{\mathrm{i}}$, ${\uptau }_{\mathrm{d}}$) are determined using closed loop oscillation tuning techniques such as the Zeigler-Nichols method and the Tyreus-Luyben method, which are outlined in Table 3.

**Table 3:** Parameters of PID Tuned Using Different Tuning Methods.

Tuning methods	${k}_{c}$	${\uptau }_{\mathrm{i}}$	${\uptau }_{\mathrm{d}}$
Zeigler-Nichols	$28.79$ 0	$14.395$ 0	$3.59$ 0
Tyreus-Luyben	$10.71$ 0	$63.33$ 0	$4.31$ 0

As a result, the PID controller that has been tuned is:

10 $${\mathrm{C}}\left( {\mathrm{s}} \right) \, = { 14}.{28} = \left( {1 + \frac{1}{14.395s} + 3.59s} \right)$$

The Z-N tuning method is engaged to adjust the three parameters of a PID controller. The initial PID controller values determined by all methods should be regularly fine-tuned through computer simulations until the closed system’s desired response is realized. This drives the enhancement of advanced and smart instruments that can help engineers in reaching the optimal performance of the PID controller.

**Fig. 4:**
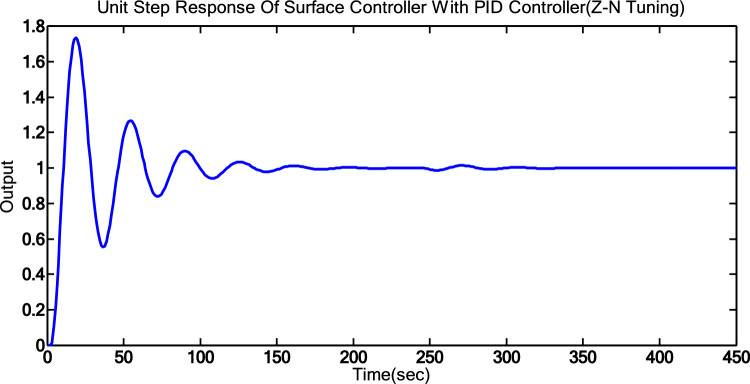
Step response for PID-controlled surface condenser using Z-N tuning.

The result indicates a significant value of 73%, which is not acceptable in a process plant. To reduce the maximum overshoot and settling time further, PID tuning is performed using Genetic algorithm. Figure 4 presents the step response for the PID-controlled surface condenser using Z-N tuning.

#### Genetic algorithm

Genetic Algorithms (GA) utilize the idea of survival of the fittest to discover optimal solutions through several iterations. As a result, GA is mainly applied to optimization challenges that focus on maximization.

##### Objective function (OBF)

In a heuristic optimization strategy based controller, the first step is to define the OBF according to the required specifications. The ITAE foundation is effective in reducing settling time, a goal that IAE or ISE based tuning is unable to achieve. Additionally, the ITAE foundation also minimizes peak overshoot. A controller based on ITSE responds significantly to sudden changes in the set point, which is not ideal from a controller design standpoint. It has been noted that ITAE serves as a superior objective function. The parameters of the PID controller are adjusted using the ITAE OBF in GA.

OBF = min (ITAE).

##### Parameters of GA

Maximum population: 30.

Maximum generations: 40.

Error criteria = ITAE.

No of variables: 3

**Fig. 5:**
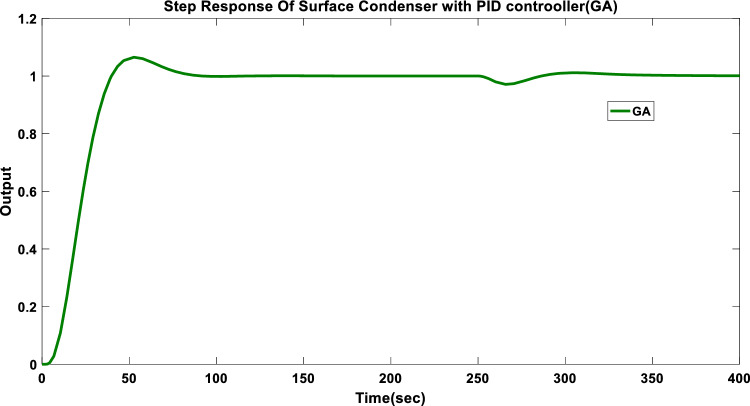
Step responses for GA based PID controller.

The output indicates overshoot of 6.5% and a settling time of 92 s. Figure 5 displays the step responses for the surface condenser controlled by a GA-Based PID controller.

#### MVO algorithm

The MVO algorithm is a population-centric computation that draws inspiration from stochastic processes. The algorithm’s approach is divided into two stages: exploration and exploitation. In the MVO algorithm, the concepts of black holes and white holes are employed to investigate the search spaces, while the concept of wormholes is utilized for exploiting those spaces. During the process of optimization, the subsequent principles are employed regarding the universes of MVO:

1. A higher inflation rate corresponds to an increased likelihood of the existence of white holes.
2. A higher inflation rate results in a decreased likelihood of the existence of black holes.
3. Universes characterized by a higher inflation rate tend to transmit variables through white holes.
4. Universes with a lower inflation rate are more likely to receive additional variables through black holes.
5. Entities in every universe can travel freely to the optimal universe through wormholes.

**Fig. 6:**
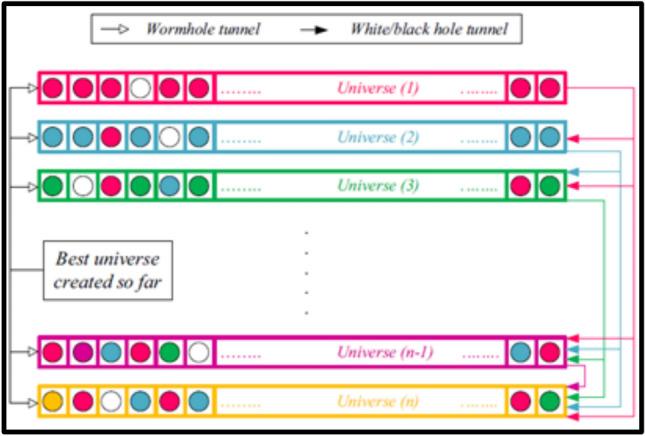
Conceptual prototype of the proposed MVO algorithm.

The theoretical model of the approached algorithm is represented in Fig. 6.

A roulette wheel system is utilized to illustrate the transfer of variables between universes through the concept of a white/black hole. In each rotation, the universes are organized according to their rates of inflation, and one is selected by this method to represent a white hole.

Assume that $$U = \left[ {\begin{array}{*{20}c} {x_{1}^{1} } & {x_{1}^{2} } & \ldots & {x_{1}^{m} } \\ {x_{2}^{1} } & {x_{2}^{2} } & \ldots & {x_{2}^{m} } \\ \vdots & \vdots & \vdots & \vdots \\ {x_{n}^{1} } & {x_{n}^{2} } & \ldots & {x_{n}^{m} } \\ \end{array} } \right]$$.

Where $$\mathrm{m}=\text{no of objects }(\mathrm{variables}$$),

$$\mathrm{n}=\text{no of universes }(\mathrm{solutions})$$

11 $$x_{i}^{j} = \left\{ {\begin{array}{*{20}c} {x_{k}^{j} r1 < NI\left( {Ui} \right)} \\ {x_{i}^{j} r1 \ge NI\left( {Ui} \right)} \\ \end{array} } \right.$$

where $${{x}_{i}}^{j}=\text{jth parameter of ith universe}$$,

$$Ui=\text{ ith universe},$$

$$NI\left(Ui\right)=\text{normalized inflation rate of ith universe}$$,

$$\mathrm{r}1\text{ is a random number}\in (\mathrm{0,1})$$ and

$${{x}_{k}}^{j}=\text{jth parameter of kth universe}$$

The exploration can ensured utilizing the roulette wheel mechanism. With a specific end goal to keep up the decent variety of universes and achieve exploitation, it has been consider that every universe has wormholes to transfer its variables over space haphazardly. In Fig. 6, white circles are equal to transferred objects through the wormholes. Continuously formed wormhole tunnels connect each universe to the most optimal universe developed to date, allowing for localized modifications in every universe and increasing the likelihood of enhancing the inflation rate through the use of wormholes as detailed below:

12 $$x_{i}^{j} = \left\{ {\begin{array}{*{20}c} {X_{j} + TDR \times \left( {\left( {ub_{j} - lb_{j} } \right) \times r4 + lb_{j} } \right) } & {r3 < 0.5} & {} \\ {X_{j} - TDR \times \left( {\left( {ub_{j} - lb_{j} } \right) \times r4 + lb_{j} } \right)} & {r3 \ge 0.5} & { r2 \ge WEP} \\ {x_{i}^{j} } & {} & {r2 < WEP} \\ \end{array} } \right.$$

where, X_j_ = ,j^th^ parameter of the best universe constructed so far.

Wormhole existence probability (WEP) = coefficient,

Travelling distance rate (TDR) = coefficient,

$${\mathrm{ub}}_{\text{j }}=\text{upper bound of }j\text{th variable},$$

$${\mathrm{lb}}_{\text{j }}=\text{lower bound of }j\text{th variable},$$

$${{x}_{i}}^{j}=\text{jth parameter of ith universe}$$

$$r2, r3 \mathrm{and} r4\text{ are random number}\in (\mathrm{0,1})$$

WEP is characterized as the possibility of wormholes presences in the universes. It is incremented linearly to amplify the local search during the optimization method.

13 $${WEP = WEP}_{{{min}}} \times \left( {\frac{{{WEP}_{{{max}}} - {WEP}_{{{min}}} }}{{L}}} \right)$$

where $${\mathrm{WEP}}_{\mathrm{min}}=0.2 \left(\mathrm{considered}\right)$$,

$${\mathrm{WEP}}_{\mathrm{max}}=1 (\mathrm{considered})$$,

$$\mathrm{l}=\mathrm{time}\left(\text{current iteration}\right)\text{ and}$$

$$\mathrm{L}=\text{maximum time}(\text{maximum iteration})$$

TDR is defined as the rate at which a variable can be transferred between a universe and the optimal universe constructed to date using wormholes. To achieve precise local search around the optimal universe, TDR is reduced over iterations.

14 $$TDR = 1 - \frac{{l^{{{1 \mathord{\left/ {\vphantom {1 p}} \right. \kern-0pt} p}}} }}{{ L^{{{1 \mathord{\left/ {\vphantom {1 p}} \right. \kern-0pt} p}}} }}$$

where, *p* illustrates the local search precision over the cycles.

#### Modified MVO (MMVO)

The MVO is likely to become stuck in local optima and to enhance the MVO’s global search capabilities and its ability to break free from nearby optima, the algorithm has been modified. The TDR value is raised throughout the cycles to enable a more accurate exploration/local search in the vicinity of the optimal universe achieved. In the MMVO algorithm, the TDR equation has been altered.

15 $$TDR = 1 - \frac{{l^{{{\raise0.7ex\hbox{$2$} \!\mathord{\left/ {\vphantom {2 p}}\right.\kern-0pt} \!\lower0.7ex\hbox{$p$}}}} }}{{ L^{{{\raise0.7ex\hbox{$2$} \!\mathord{\left/ {\vphantom {2 p}}\right.\kern-0pt} \!\lower0.7ex\hbox{$p$}}}} }}$$

**Fig. 7:**
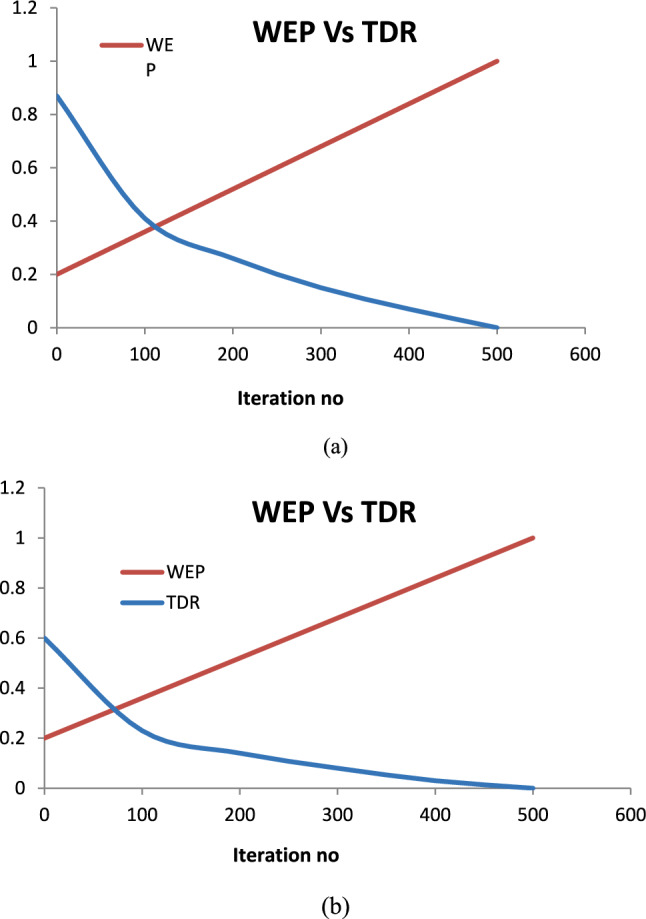
(**a**) WEP versus TDR of MVO Algorithm (**b**) WEP versus TDR of MMVO Algorithm.

WEP and TDR are demonstrated in Fig. 7.

**Fig. 8:**
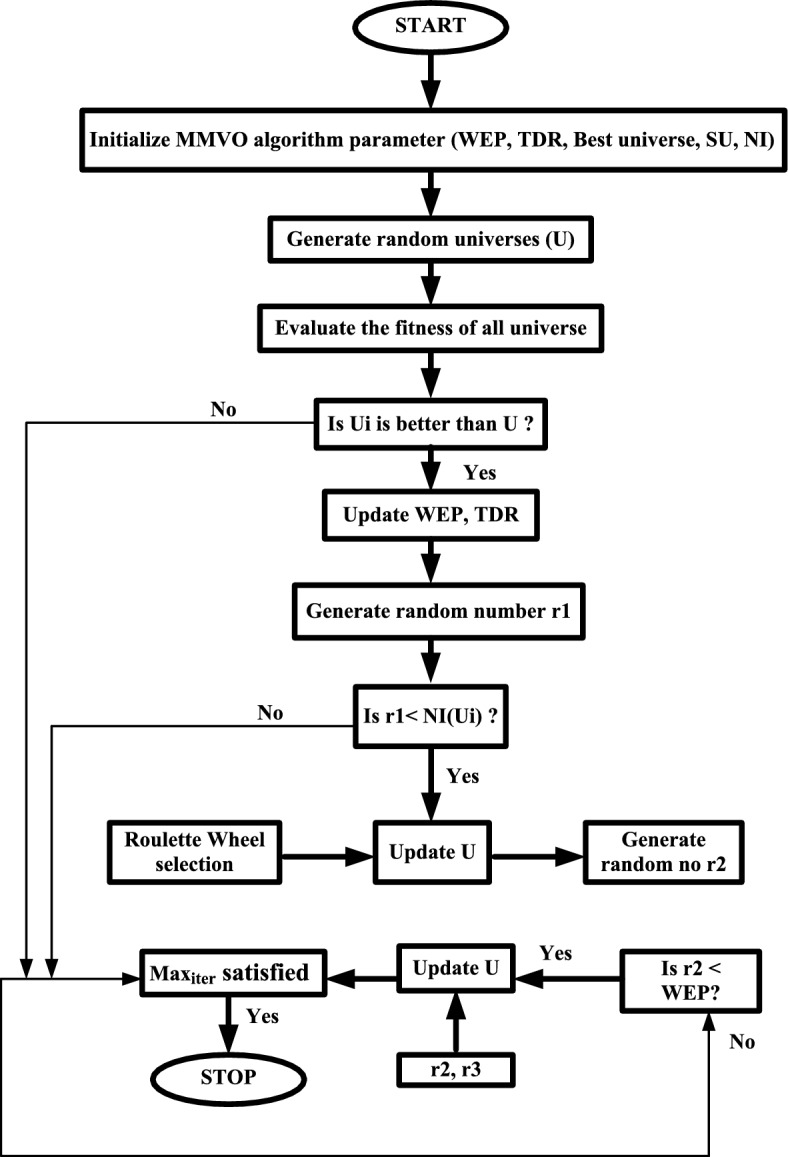
Flow chart of MMVO algorithm.

To isolate the contribution of the modified Travelling Distance Rate (TDR) schedule in MMVO, an ablation-style comparison was carried out. In this study, all algorithmic settings including population size, number of iterations, parameter bounds, WEP schedule, and initialization were kept identical, and only the TDR formulation was switched between the original MVO and the modified MMVO. This controlled comparison demonstrated that the improvement achieved by MMVO is primarily attributable to the revised TDR update. For clarity comparative plot has been included to illustrate their variation across iterations. In the standard MVO, the TDR value decreases gradually to promote local exploitation in later iterations, whereas in MMVO, the TDR is increased progressively to enhance exploration around promising regions. This distinction in TDR dynamics provides the underlying mechanism that enables MMVO to escape local optima more effectively (Fig. 8).

The MMVO algorithm is employed to adjust the gains of the PID controller to increase the performance under standard operating conditions, searches for the optimal combination of these parameters by minimizing an ITAE-based objective function, selected for its ability to penalize prolonged error and overshoot. At each iteration, candidate solutions (universes) are updated through the MMVO’s modified wormhole and TDR mechanisms, allowing exploration of the search space and refinement around promising regions. The process continues until convergence criteria are met, resulting in a set of PID gains that yield improved dynamic performance for the condenser system. The optimized parameters are kp = 2.3866, ki = 0.0538 and Kd = 10.0000.

## Results and comparisons

To justify the efficiency of the proposed MMVO approach, 23 test functions are selected. They are exceptional and have been extensively grasped by various examiners. The standard benchmark functions are recorded in Tables 4, 5 and 6 where ${\mathrm{f}}_{\mathrm{min}}$ illustrate the optimal value of the function. Except ${\mathrm{f}}_{8},$ the test functions starting from ${\mathrm{f}}_{1 }-{\mathrm{f}}_{13}$ has value of ${\mathrm{f}}_{\mathrm{min}}$ is zero. Function ${\mathrm{f}}_{14}-{\mathrm{f}}_{23}$ has nonzero ${\mathrm{f}}_{\mathrm{min}}$ values. The test functions can be isolated into three gatherings:

$${\mathrm{f}}_{1}-{\mathrm{f}}_{8}=\text{unimodal range of benchmark functions}$$ ^28^

$${\mathrm{f}}_{8}-{\mathrm{f}}_{13}=\text{multimodal range of benchmark functions}$$ ^29^

$${\mathrm{f}}_{14}-{\mathrm{f}}_{23}=\text{fixed dimension range of benchmark functions}$$ [30].

### Analysis of unimodal benchmark functions

The dimensions of the benchmark functions f_1_–f_13_ were kept at 5, 10, 20, and 30. The maximum number of iterations was set to 500, and the number of universes was fixed at 30. Each algorithm was executed 30 independent times to enable statistical analysis, and the best, worst, mean, and standard deviation values were computed for all runs. For verification purposes, the performance of the proposed MMVO algorithm was compared with that of the standard MVO. The results for the unimodal benchmark functions f_1_–f_8_ are presented in Table 7. From these results, it can be observed that the unimodal functions are well suited for evaluating local search capability, and the MMVO algorithm demonstrates superior performance compared to MVO.

**Table 7:** Results of unimodal benchmark functions.

Function (Dim)	Optimizer	Best Value	Worst Value	Avg Value	Standard deviation
F_1_(5)	MVO	0.0001	0.0021	0.0009	0.0005
	MMVO	10^− 4^*0.0437	10^− 4^*0.9831	10^− 4^*0.3005	10^− 4^*0.2019
F_1_(10)	MVO	0.0059	0.0468	0.0159	0.0091
	MMVO	10^− 3^*0.1630	10^− 3^*0.9128	10^− 3^*0.3834	10^− 3^*0.1641
F_1_(20)	MVO	0.1205	0.3998	0.2552	0.0672
	MMVO	0.0025	0.0099	0.0066	0.0020
F_1_(30)	MVO	0.1231	0.4994	0.2537	0.0889
	MMVO	0.0031	0.0132	0.0064	0.0023
F_2_(5)	MVO	0.0033	0.0114	0.0067	0.0020
	MMVO	0.0005	0.0033	0.0012	0.0006
F_2_(10)	MVO	0.0026	0.0146	0.0056	0.0025
	MMVO	0.0004	0.0017	0.0010	0.0003
F_2_ (20)	MVO	0.0030	0.0086	0.0058	0.0017
	MMVO	0.0005	0.0020	0.0011	0.0004
F_2_ (30)	MVO	0.0025	0.0124	0.0060	0.0022
	MMVO	0.0006	0.0023	0.0011	0.0004
F_3_(5)	MVO	10^3^*3.4295	10^3^*9.1155	10^3^*5.9670	10^3^*1.3709
	MMVO	10^3^*1.2005	10^3^*2.8681	10^3^*1.0427	10^3^*0.0129
F_3_(10)	MVO	0.0129	0.2513	0.1023	0.0659
	MMVO	0.0004	0.0071	0.0025	0.0018
F_3_(20)	MVO	0.0148	0.3312	0.1111	0.0742
	MMVO	0.0008	0.0085	0.0036	0.0018
F_3_(30)	MVO	95.9624	539.2682	212.6539	101.6505
	MMVO	4.8616	33.6298	15.6809	7.1723
F_4_(5)	MVO	0.0092	0.0414	0.0210	0.0064
	MMVO	0.0019	0.0064	0.0036	0.0012
F_4_(10)	MVO	0.0436	0.1968	0.0936	0.0348
	MMVO	0.0053	0.0246	0.0156	0.0051
F_4_(20)	MVO	0.2524	1.2720	0.5299	0.2203
	MMVO	0.0465	0.2475	0.0934	0.0465
F_4_(30)	MVO	0.2853	1.6137	0.6114	0.3253
	MMVO	0.0443	0.2058	0.0860	0.0334
F_5_(5)	MVO	0.0209	218.8522	11.1746	39.4784
	MMVO	0.0863	345.3149	27.4791	78.8079
F_5_(10)	MVO	5.0332	917.1020	100.5175	199.3305
	MMVO	0.5740	793.4167	102.8266	182.9023
F_5_(20)	MVO	10^3^*0.0180	10^3^*2.7793	10^3^*0.5887	10^3^*0.9497
	MMVO	10^3^*0.0119	10^3^*1.7405	10^3^*0.3186	10^3^*0.5285
F_5_(30)	MVO	10^3^*0.0417	10^3^*2.5880	10^3^*0.4284	10^3^*0.5492
	MMVO	10^3^*0.0214	10^3^*2.1279	10^3^*0.3091	10^3^*0.4918
F_6_(5)	MVO	0.0002	0.0026	0.0010	0.0005
	MMVO	10^− 4^*0.0295	10^− 4^*0.6011	10^− 4^*0.2999	10^− 4^*0.1551
F_6_(10)	MVO	0.0038	0.0508	0.0166	0.0096
	MMVO	10^− 3^*0.1539	10^− 3^*0.9812	10^− 3^*0. 4028	10^− 3^*0.1976
F_6_(20)	MVO	0.0742	0.4881	0.2473	0.0826
	MMVO	0.0034	0.0148	0.0073	0.0029
F_6_(30)	MVO	0.4540	2.2909	1.2636	0.3915
	MMVO	0.0218	0.0678	0.0356	0.0118
F_7_(5)	MVO	0.0001	0.0039	0.0011	0.0010
	MMVO	0.0001	0.0048	0.0015	0.0012
F_7_(10)	MVO	0.0006	0.0092	0.0030	0.0020
	MMVO	0.0013	0.0098	0.0044	0.0023
F_7_(20)	MVO	0.0039	0.0293	0.0163	0.0072
	MMVO	0.0056	0.0284	0.0144	0.0052
F_7_(30)	MVO	0.0139	0.0665	0.0334	0.0123
	MMVO	0.0126	0.0517	0.0305	0.0083

### Analysis of multi-modal benchmark functions

The multi-modal test function includes a global optimum, while the number of local optima rises exponentially as the dimensionality rises. It serves well for assessing the exploratory abilities of different algorithms. The results of benchmark functions ${f}_{8}$-${f}_{13}$. for various methods are demonstrated in Table 8. The experimental analysis illustrates that the MMVO performs particularly well in terms of exploration and in escaping undesirable local optima.

**Table 8:** Results of multimodal benchmark functions.

Function(Dim)	Optimizer	Best Value	Worst Value	Avg Value	Standard Deviation
F_8_(5)	MVO	10^3^*− 1.9765	10^3^*− 1.4040	10^3^*− 1.6973	10^3^*0.1558
	MMVO	10^3^*− 1.9765	10^3^*− 1.1473	10^3^*− 1.6651	10^3^*0.1133
F_8_(10)	MVO	10^3^*− 3.4595	10^3^*− 2.3113	10^3^*− 2.9420	10^3^*0.2930
	MMVO	10^3^*− 3.7358	10^3^*− 2.4313	10^3^*− 2.9543	10^3^*0.0396
F_8_(20)	MVO	10^3^*− 6.9775	10^3^*− 4.1524	10^3^*− 5.5929	10^3^*0.6782
	MMVO	10^3^*− 6.8399	10^3^*− 4.1338	10^3^*− 5.4966	10^3^*0.6573
F_8_(30)	MVO	10^3^*− 9.0881	10^3^*− 6.1626	10^3^*− 7.6955	10^3^*0.6722
	MMVO	10^3^*− 9.7463	10^3^*− 6.8801	10^3^*− 7.8512	10^3^*0.7027
F_9_(5)	MVO	0.0001	2.9865	1.2276	0.8132
	MMVO	2.9849	33.8285	15.0239	7.0613
F_9_(10)	MVO	6.9774	42.7912	18.4136	8.0775
	MMVO	18.9043	78.6014	37.5432	15.5503
F_9_ (20)	MVO	24.0074	108.5279	64.5982	20.3457
	MMVO	45.7708	160.1910	95.2538	26.9696
F_9_ (30)	MVO	65.1016	203.6829	123.1528	37.4187
	MMVO	85.5832	234.8331	163.0262	37.6935
F_10_(5)	MVO	0.0079	0.0398	0.0179	0.0074
	MMVO	0.0014	0.0041	0.0027	0.0007
F_10_(10)	MVO	0.0350	2.5927	0.6586	0.7950
	MMVO	0.0051	2.0135	0.0748	0.3662
F_10_(20)	MVO	0.1281	2.2181	1.0549	0.7047
	MMVO	0.0146	17.5375	0.7859	3.2006
F_10_(30)	MVO	0.6150	3.1470	1.7065	0.5775
	MMVO	0.0376	19.0249	4.6842	7.3763
F_11_(5)	MVO	0.0221	0.3327	0.1284	0.0803
	MMVO	0.0100	0.1433	0.0602	0.0283
F_11_(10)	MVO	0.1462	0.6980	0.3957	0.1468
	MMVO	0.1197	0.5143	0.2745	0.1007
F_11_(20)	MVO	0.3035	0.7756	0.5230	0.1296
	MMVO	0.0143	0.1419	0.0642	0.0273
F_11_(30)	MVO	0.6109	0.9375	0.8453	0.0719
	MMVO	0.0669	0.3392	0.1511	0.0611
F_12_(5)	MVO	10^− 3^*0.0131	10^− 3^*0.4604	10^− 3^*0.0925	10^− 3^*0.0981
	MMVO	0.0000	3.1151	0.3113	0.7973
F_12_(10)	MVO	0.0001	1.2741	0.0785	0.2510
	MMVO	0.0000	8.5181	1.5847	2.3329
F_12_(20)	MVO	0.0021	2.6268	0.9663	0.8879
	MMVO	0.0001	7.4979	4.0245	1.9826
F_12_(30)	MVO	0.1193	9.1042	2.0443	1.8611
	MMVO	1.8271	9.3383	5.4021	1.7861
F_13_(5)	MVO	0.0000	0.0017	0.0002	0.0003
	MMVO	10^− 4^*0.0026	10^− 4^*0.2089	10^− 4^*0.0724	10^− 4^*0.0629
F_13_(10)	MVO	0.0009	0.0190	0.0082	0.0061
	MMVO	0.0000	0.0111	0.0004	0.0020
F_13_(20)	MVO	0.0223	0.1589	0.0576	0.0253
	MMVO	0.0016	0.0334	0.0099	0.0080
F_13_(30)	MVO	0.0723	0.5130	0.2187	0.1053
	MMVO	0.0016	0.0334	0.0099	0.0080

### Analysis on fixed-dimension multimodal benchmark functions

In comparison to functions f_8_–f_13_, the functions f_14_ –f_23_ are more straightforward due to their reduced dimensionality and a smaller number of local minima. The performance of benchmark functions f_14_ through f_23_ across various algorithms is presented in Table 9. The functions f_14_–f_23_ are capable of easily achieving the global optimum. According to Table 9, it is evident that MMVO yields superior solutions compared to other algorithms, with the exceptions of f_14_ and f_18._

**Table 9:** Outcomes of benchmark functions with fixed dimensions across multiple modalities.

Function(Dim)	Optimizer	Best value	Worst value	Avg value	Standard deviation
F_14_(2)	MVO	0.9980	0.9980	0.9980	0.0000
	MMVO	0.9980	20.1535	9.1525	6.1129
F_15_(4)	MVO	0.0007	0.0631	0.0095	0.0137
	MMVO	0.0003	0.0204	0.0052	0.0079
F_16_(2)	MVO	− 1.0316	− 1.0316	− 1.0316	0.0001
	MMVO	− 1.0316	− 0.2155	− 0.9500	0.0000
F_17_(2)	MVO	0.3979	0.3979	0.3979	0.0000
	MMVO	0.3979	0.3979	0.3979	0.0000
F_18_(2)	MVO	3.0000	84.0000	8.4000	20.5504
	MMVO	3.0000	84.0000	16.5000	25.3183
F_19_(3)	MVO	− 3.8628	− 3.8628	− 3.8628	0.0000
	MMVO	− 3.8628	− 3.8628	− 3.8628	0.1411
F_20_(6)	MVO	− 3.3220	− 3.1965	− 3.2817	0.0579
	MMVO	− 3.3220	− 3.2025	− 3.2862	0.0556
F_21_(4)	MVO	− 10.1531	− 2.6304	− 7.2943	3.0109
	MMVO	− 10.1531	− 2.6304	− 5.5583	3.2217
F_22_(4)	MVO	− 10.4028	− 1.8376	− 7.9625	3.3542
	MMVO	− 10.4028	− 1.8376	− 6.1228	3.0514
F_23_(4)	MVO	− 10.5364	− 2.4273	− 8.5842	3.1123
	MMVO	− 10.5364	− 1.6766	− 4.3841	3.0223

### Comparison of statistical result with MFO

Performance of the MMVO is compared with the MFO in terms of best value for 40 dimensions, 60 no of universe and 600 iteration. For the comparison involving the Moth-Flame Optimization (MFO) algorithm, the experimental settings were intentionally selected to differ from those used in the evaluations of MVO and MMVO. MFO has been reported in prior literature to achieve its most stable performance when higher dimensionality, larger population sizes, and increased iteration limits are employed. Tables 11 and 12 shows the statistical results of unimodal and multimodal benchmark functions respectively.

From Table 10, it can be observed that the MMVO outperforms than MFO except in F_3_.

**Table 10:** Comparison result of unimodal benchmark function with MFO.

Function	MMVO (Best Value)	MFO (Best Value)
F_1_	0.0210	2.253
F_2_	0.0946	0.6584
F_3_	0.9115	${10}^{3}$*9.8165
F_4_	0.2856	51.6426
F_5_	${10}^{3}$*0.328	549.6379
F_6_	0.0210	3.4127
F_7_	0.0082	0.9999

From Table 11, it can be observed that the MMVO gives satisfactory result than MFO.

**Table 11:** Comparison result of multimodal benchmark function with MFO.

Function	MMVO(Best Value)	MFO(Best Value)
F_8_	10^4^*− 1.2512	10^4^*− 1.358
F_9_	112.4057	117.6214
F_10_	0.0327	1.1029
F_11_	0.0827	1.0064
F_12_	1.8754	3.7687
F_13_	0.0017	17.2521

### Simulation results

**Fig. 10:**
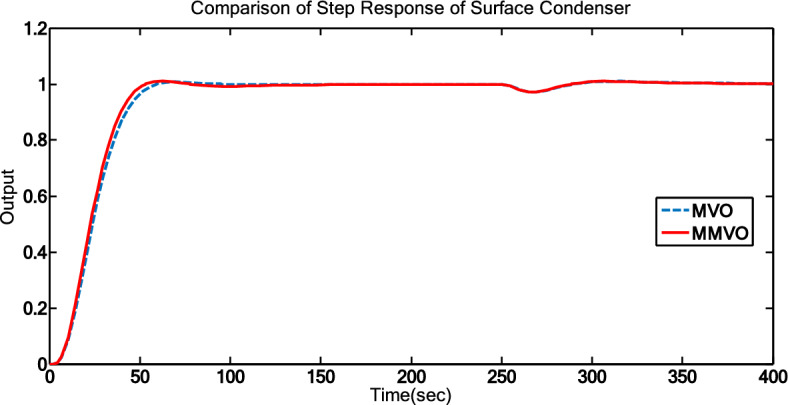
Step Responses of MVO and MMVO.

**Fig. 11:**
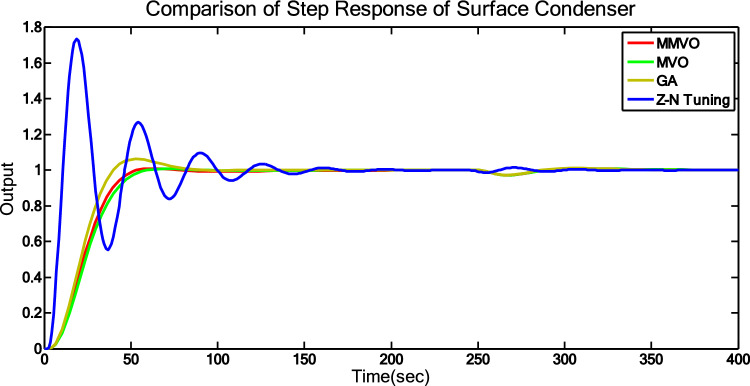
Step responses of all methods.

The effectiveness of various techniques is assessed through time response analysis and standard error metrics. The step response analysis for the optimization methods (MVO and MMVO) can be seen in Fig. 10, while the step response analysis for all methods is presented in Fig. 11. To ensure consistency between the numerical results and the graphical responses, the controller parameters and operating conditions used in Figs. 10 and 11 have been directly aligned with those presented in Tables 12, 13 and 14. The simulations were executed over several independent runs for each tuning method. Across these runs, the time-response characteristics exhibited stable and repeatable trends, with no significant deviation from the representative responses shown in the figures. The displayed curves therefore reflect the typical system behavior observed during the repeated trials, and their correspondence with the tabulated performance indices confirms the reliability of the comparative analysis. Tables 12, 13 and 14 outline the controller parameters, performance metrics, and error criteria, respectively. To evaluate performance, peak time, maximum overshoot, and settling time are calculated. The reduction of error is achieved by employing ISE, IAE, ITSE, and ITAE criteria.

**Table 12:** PID controller parameters using various tuning techniques.

Tuning method	${K}_{P}$	${K}_{i}$	${K}_{d}$
Z-N Tuning	14.28	0.9920	51.26
GA	2.713	0.067	9.903
MVO	2.1854	0.0514	9.9194
MMVO	2.3866	0.0538	10.0000

**Table 13:** Comparison of different parameters indices.

Parameters	MMVO	MVO	PID(GA)	PID(Z-N Tuning)
ITSE	194.6	215.2	225.2	243.1
ITAE	759.2	783.4	801.3	1141
ISE	16.85	17.7	17.5	12.81
IAE	24.75	25.96	27.21	27.88

**Table 14:** Analysis of time response parameters.

Parameters	MMVO	MVO	PID(GA)	PID(Z-N Tuning)
Settling Time(s)	53	60	92	152
Maximum Peak Overshoot (%)	1	0.9	6.5	73.5
Peak Time(s)	1.01	1.009	1.065	1.735

16 $$IAE={\int }_{0}^{\infty }\left|e(t)\right| dt$$

17 $$ISE={\int }_{0}^{\infty }{e}^{2}\left(t\right) dt$$

18 $$ITAE ={\int }_{0}^{\infty }t \left|e(t)\right| dt$$

19 $$ITSE = {\int }_{0}^{\infty }{t e}^{2}\left(t\right) dt$$

MMVO-based optimization yields satisfactory outcomes with reduced overshoot and settling time, even though the rise time is somewhat longer compared to MVO optimization. The error factor are also reduced in the MMVO optimization. The proposed MMVO algorithm was found to deliver the lowest ITAE, ITSE, and IAE values, whereas the Ziegler–Nichols method resulted in a smaller ISE under the present operating conditions. As a result, Fig. 11 indicates that the response obtained with the MMVO-tuned PID controller is improved in comparison with the other methods.

The PID controller utilizing the MMVO algorithm yields the lowest error indices.

From Table 14, it is observed that the tuning of PID using Z-N Tuning method shows high overshoot on the range of 73.5% and settling time 152s. To compensate the high peak overshoot, PID controller tuned using GA. It gives 6.5% peak overshoot and 92s settling time. PID controller tuned using MVO algorithm shows settling time 60s. Then three parameters of PID are tuned by using MMVO algorithm. In this case, it takes 53s to settle down and shows overshoot in the range of 1%.

## Conclusion and future scope

This paper examines the performance of a PID controller using various tuning methods and optimization techniques. The main objective is to maintain the temperature of the outward liquid of a gas to liquid type warmth exchanger framework to a coveted temperature in the less conceivable time and least or no overshoot independent of changes in the load and process disturbances. Based on the observations, it’s evident that the PID controller optimized with MMVO delivers impressive outcomes with reduced overshoot and settling time, outperforming the PID controller tuned via the Z-N method, as well as the GA-tuned PID parameter optimization. PID controller with MMVO optimization offers overshoot in the limit of 1% and settling time 53s. According to the best value, worst value, standard deviation and average value of the benchmark functions, MMVO algorithm outperforms than other methods. Although the PID controller with MMVO optimization technique gives better result than that of the tuning of PID controller by Z-N method, but improvised controller with improvised optimization technique will be used to handle the system with increase in nonlinearities.

This study has several limitations that define its current scope. The condenser system is modeled using a simplified process representation, and real plant nonlinearities such as fouling, varying steam quality, and measurement noise are not fully captured. The optimization is performed using a single ITAE-based objective function, and multi-objective trade-offs were not explored. In addition, the evaluation is limited to simulation results, and no hardware or real-time validation has been conducted. The performance of the proposed MMVO method has been assessed under fixed operating conditions, and its adaptability to rapidly changing industrial scenarios remains to be investigated. These limitations indicate opportunities for further development and experimental verification.

## Data Availability

No datasets were generated or analysed during the current study.

## Appendix

See Tables 4, 5 and 6.

**Table 4:** Unimodal test functions.

Function	Range	${f}_{min}$
$F_{1} \left( x \right) = \mathop \sum \limits_{i = 1}^{n} x_{i}^{2}$	[− 100,100]	0
$F_{2} \left( x \right) = \mathop \sum \limits_{i = 1}^{n} \left| {x_{i} } \right| + \mathop \prod \limits_{i = 1}^{n} \left| {x_{i} } \right|$	[− 10,10]	0
$F_{3} \left( x \right) = \mathop \sum \limits_{i = 1}^{n} \left( {\mathop \sum \limits_{j - 1}^{n} x_{j} } \right)^{2}$	[− 100,100]	0
$F_{4} \left( x \right) = max_{i } \left\{ {\left| {x_{i} } \right|, 1 \le i \le n} \right\}$	[− 100,100]	0
$F_{5} \left( x \right) = \mathop \sum \limits_{i = 1}^{n} \left[ {100\left( {x_{i + 1} - x_{i}^{2} } \right)^{2} + \left( {x_{i} - 1} \right)^{2} } \right]$	[− 30,30]	0
$F_{6} \left( x \right) = \mathop \sum \limits_{i = 1}^{n} \left( {\left[ {x_{i} + 0.5} \right]} \right)^{2}$	[− 100,100]	0
$F_{7} \left( x \right) = \mathop \sum \limits_{i = 1}^{n} ix_{i}^{4} + {\mathrm{random}}[0,1]$	[− 1.28,1.28]	0

**Table 5:** Multi-modal benchmark functions.

Function	Range	${f}_{min}$
$F_{8} \left( x \right) = \mathop \sum \limits_{i = 1}^{n} - x_{i} {sin}\left( {\sqrt {\left| {x_{i} } \right|} } \right)$	[− 500,500]	0
$F_{9} \left( x \right) = \mathop \sum \limits_{i = 1}^{n} \left[ {x_{i}^{2} - 10{cos}\left( {2\pi x_{i} } \right) + 10} \right]$	[− 5.12.5.12]	0
$$\begin{gathered} F_{10} \left( x \right) = - 20{exp}\left( { - 0.2\sqrt {\frac{1}{n}\mathop \sum \limits_{i = 1}^{n} x_{i}^{2} } } \right) \hfill \\ - { exp}\left( {\frac{1}{n}\mathop \sum \limits_{i = 1}^{n} {cos}\left( {2\pi x_{i} } \right)} \right)20 + e \hfill \\ \end{gathered}$$	[− 32,32]	0
$F_{11} \left( x \right) = \frac{1}{4000}\mathop \sum \limits_{i = 1}^{n} x_{i}^{2} - \mathop \prod \limits_{i = 1}^{n} {cos}\left( {\frac{{x_{i} }}{\sqrt i }} \right) + 1$	[− 600,600]	0
$$\begin{gathered} F_{12} \left( x \right) = \frac{\pi }{n}\left\{ {10{sin}\left( {\pi y_{1} } \right) + \mathop \sum \limits_{i = 1}^{n} \left( {y_{i} - 1} \right)^{2} \left[ {1 + 10{sin}^{2} \left( {\pi y_{i + 1} } \right) + \left( {y_{n} - 1} \right)^{2} } \right]} \right\} \hfill \\ + \mathop \sum \limits_{i = 1}^{n} u\left( {x_{i } ,10,100,4} \right) \hfill \\ \end{gathered}$$ $y_{i} = 1 + \frac{{x_{j} + 1}}{4}$ $$u\left( {x_{i } ,a,k,m} \right) = \left\{ {\begin{array}{*{20}c} {k\left( {x_{i} - a} \right)^{m} x_{i} > a} \\ {0 - a < x_{i} < a} \\ {k\left( { - x_{i} - a} \right)^{m} x_{i} < - a} \\ \end{array} } \right.$$	[− 50,50]	0
$$\begin{gathered} F_{13} \left( x \right) = 0.1\left\{ {{sin}^{2} \left( {3\pi x_{1} } \right) + \mathop \sum \limits_{i = 1}^{n} \left( {x_{i} - 1} \right)^{2} \left[ {1 + 10{sin}^{2} \left( {3\pi x_{i} + 1} \right)} \right] + \left( {x_{n} - 1} \right)^{2} \left[ {1 + {sin}^{2} \left( {2\pi x_{n} } \right)} \right]} \right\} \hfill \\ + \mathop \sum \limits_{i = 1}^{n} u\left( {x_{i } ,5,100,4} \right) \hfill \\ \end{gathered}$$	[− 50,50]	0

**Table 6:** Fixed-dimension multimodal benchmark functions.

Function	Range	${f}_{min}$
$F_{14} \left( x \right) = \left( {\frac{1}{500}\mathop \sum \limits_{j = 1}^{25} \frac{1}{{j + \mathop \sum \nolimits_{i = 1}^{2} \left( {x_{i} - a_{ij} } \right)^{6} }}} \right)$	[− 65.53, 65.53]	0.998004
$F_{15} \left( x \right) = \mathop \sum \limits_{i = 1}^{11} \left( {ai - \frac{{x_{1} \left( {b_{i}^{2} + b_{i } x_{2} } \right)}}{{b_{i}^{2} + b_{i } x_{3} + x_{4} }}} \right)^{2}$	[− 5,5]	0.0003075
$F_{16} \left( x \right) = 4x_{1}^{2} - 2.1x_{1}^{4} + \frac{1}{3}x_{1}^{6} + x_{1} x_{2} - 4x_{2}^{2} + 4x_{2}^{4}$	[− 50,50]	− 1.0316285
$F_{17} \left( x \right) = \left( {x_{2} - \frac{5.1}{{4\pi^{2} }}x_{1}^{2} + \frac{5}{\pi }x_{1} - 6} \right)^{2} + 10\left( {1 - \frac{1}{8\pi }} \right)\cos x_{1} + 10$	Lb = [− 5,10]	
Ub = [0,15]	0.398	
$$\begin{gathered} F_{18} \left( x \right) = \left[ {1 + \left( {x_{1} + x_{2} + 1} \right)\left( {19 - 14x_{1} + 3x_{1}^{2} - 14x_{2} + 6x_{1} x_{2} + 3x_{2}^{2} } \right)} \right]^{2} \hfill \\ \times \left[ {30 + \left( {2x_{1} - 3x_{2} } \right)^{2} \left( {18 - 32x_{1} + 12x_{1}^{2} + 48x_{2} - 36x_{1} x_{2} + 327^{2} } \right)} \right] \hfill \\ \end{gathered}$$	[− 5,5]	3
$F_{19} \left( x \right) = - \mathop \sum \limits_{i = 1}^{4} c_{i} {exp}\left( { - \mathop \sum \limits_{j = 1}^{3} a_{ij} \left( {x_{j} - p_{ij} } \right)^{2} } \right)$	[− 0,1]	− 3.86
$F_{20} \left( x \right) = - \mathop \sum \limits_{i = 1}^{4} c_{i} {exp}\left( { - \mathop \sum \limits_{j = 1}^{6} a_{ij} \left( {x_{j} - p_{ij} } \right)^{2} } \right)$	[− 0,1]	− 3.32
$F_{21} \left( x \right) = - \mathop \sum \limits_{i = 1}^{5} \left[ {\left( {X - a_{i} } \right)\left( {X - a_{i} } \right)^{T} + c_{i} } \right]^{ - 1}$	[0,10]	− 10.1532
$F_{22} \left( x \right) = - \mathop \sum \limits_{i = 1}^{7} \left[ {\left( {X - a_{i} } \right)\left( {X - a_{i} } \right)^{T} + c_{i} } \right]^{ - 1}$	[0,10]	− 10.4029
$F_{23} \left( x \right) = - \mathop \sum \limits_{i = 1}^{10} \left[ {\left( {X - a_{i} } \right)\left( {X - a_{i} } \right)^{T} + c_{i} } \right]^{ - 1}$	[0,10]	− 10.5364

## Abbreviations

IAE: Integral absolute error
ISE: Integral squared error
ITAE: Integral of time multiplied absolute error
ITSE: Integral of time multiplied squared error
GA: Genetic algorithm
MFO: Moth flame optimization
MVO: Multi verse optimizer
MMVO: Modified multi verse optimizer
PID: Proportional plus integral plus derivative
Z-N: Ziegler-Nichols
